# Computation of distribution of relaxation times by Tikhonov regularization for Li ion batteries: usage of L-curve method

**DOI:** 10.1038/s41598-021-91871-3

**Published:** 2021-06-16

**Authors:** T. Paul, P. W. Chi, Phillip M. Wu, M. K. Wu

**Affiliations:** 1 Institute of Physics, Academia Sinica, 115 Taipei, Taiwan; 2 BitSmart LLC, San Mateo, CA USA; 3 Present Address: Department of Materials and Mineral Resources Engineering, National Taipei University of Technology, 1, Sec. 3, Zhong-Xiao E. Rd., Taipei, 10608 Taiwan

**Keywords:** Mathematics and computing, Physics

## Abstract

In this paper, the distribution of relaxation times (DRTs) functions are calculated numerically in Matlab for synthetic impedance data from single parallel $RC$ circuit and two parallel $RC$ circuits connected in series, experimental impedance data from supercapacitors and α-LiFeO_2_ anode based Li ion batteries. The quality of the impedance data is checked with the Kramers–Krönig (KK) relations. The DRTs are calculated within the KK compatible regime for all the systems using Tikhonov regularization (TR) method. Here we use a fast and simple L-curve method to estimate the TR parameter (λ) for regularization of the Fredholm integral equations of first kind in impedance. Estimation of the regularization parameters are performed effectively from the offset of the global corner of the L-curve rather than simply using the global corner. The physical significances of DRT peaks are also discussed by calculating the effective resistances and capacitances coupled with peak fitting program. For instance, two peaks in the DRTs justify the electrical double layer capacitance and ion diffusion phenomena for supercapacitors in low to intermediate frequencies respectively. Moreover, the surface film effect, Li/electrolyte and electrode/electrolyte charge transfer related processes are identified for α-LiFeO_2_ anode based Li-ion batteries. This estimation of the offset of the global corner extends the L-curve approach coupled with the Tikhonov regularization in the field of electrochemistry and can also be applied in similar process detection methods.

## Introduction

Electrochemical impedance spectroscopy (EIS) is useful to separate contributions of different electrochemical phenomena related to polarization losses, basic ion transport and kinetic parameters of various electrochemical cells, and batteries^1–6^. EIS is often analyzed with complex non-linear least squares fit, which is computed by equivalent circuit modelling. However, equivalent circuits are not easy to find and it is generally difficult to have proper circuit for complex electrochemical phenomena.

It is of utmost interest of researchers to find alternative approach to interpret the EIS data. Distribution of relaxation times (DRT) provide a rich analysis of EIS by transforming the data from frequency ($f$) domain to time $\left( \tau \right)$ domain. DRTs exhibit peaks or single peak on a $\log \left( \tau \right)$ axis (or $f$ axis) indicating some electrochemical processes. The primary advantage of DRTs is its representation being model free and do not need much information about the system. Up to now several methods have been adopted to invert impedance data to a DRT such as Tikhonov Regularization (TR)^7–16^, Maximum Entropy (ME)^17^, Fourier transform (FT)^18–20^. Concerning TR, ridge and Lasso methods need an adjustment of the regularization parameters to get reasonable DRTs and different values lead to different number of peaks hindering true electrochemical phenomena^21^. The DRTs of yttria and calcium stabilized cubic zirconia have been computed by choosing regularization parameter based on root mean square error of impedance^22^ as well as by least square method coupled with Tikhonov regularization^15^. Combination of Tikhonov regularization and multi-(RQ) CNLS-fits technique is used for solid oxide fuel cells and steam electrolysers to determine the analytic form of DRTs^18^. DRTs based on ME method has been computed for NASICON materials^17^. Both ionic and electronic contribution are determined from DRTs of solid oxide fuel cell cathodes by Fourier transformation of the EIS data^23^. Least Absolute Shrinkage and Selection Operator^21^ based regularization and multi-(RQ) CNLS-fits^24,25^ are used for commercial Li-ion batteries for EIS data synergistically to compute the DRTs. On the other hand, another approach has been made by the group of Tsur^26–28^ by constructing a parametric form of DRTs. The advantage of this method is, it does not need any smoothing parameter or window function. DRTs based on this approach have been computed for oxide ion conducting electrolytes^29^ of solid oxide fuel cells, ceramic actuators^30^ as well as Fe and Ni based catalysts^31^.

The goal of this work is to provide a simple and fast method to calculate DRTs using Matlab application with valid regularization parameter which is determined by L-curve. The offset of the global corner of the L-curve method leads to better estimate results for all the studied examples. To demonstrate this new method, we have worked on the synthetic impedance data from single unit of $RC$ circuit as well as series connection of $RC$ circuits. DRTs are also calculated for graphite-based supercapacitors and LiFeO_2_ anode based Li-ion batteries. A Matlab based curve fitting program is also used to estimate the $R$ and $C$’s from the DRTs quantitatively.

## Method of simulation

The data inversion in calculating DRTs needs to solve a Fredholm integral equation of first kind supposing the impedance spectrum as $Z^{*} \left( \omega \right) = Z^{\prime}\left( \omega \right) - jZ^{\prime\prime}\left( \omega \right)$, where $Z^{*} \left( \omega \right)$ is the complex impedance and $Z^{\prime}$ and $Z^{\prime\prime}$ are the real and imaginary parts of complex impedance, respectively. Equation (1) as per Ref.^32^ presents the relation between impedance data and its frequency distribution,

1 $$ Z^{*} = R_{\infty } + R_{p} \mathop \smallint \limits_{ - \infty }^{ + \infty } \frac{{G\left( {\log \left( \tau \right)} \right)}}{1 + j\omega \tau }d\left( {\log \left( \tau \right)} \right), $$

where $R_{\infty }$ is the series resistance, $R_{p}$ is the total polarization resistance, $G$ is the DRT and $\tau = RC$ is the relaxation time,$R$ is the effective resistance and $C$ is the effective capacitance, and $\omega$ is the angular frequency. Considering $\omega = 0$ the normalization condition can be obtained as $\mathop \smallint \limits_{ - \infty }^{ + \infty } G\left( {\log \left( \tau \right)} \right)d\left( {\log \left( \tau \right)} \right) = 1$. Decomposing both real and imaginary parts of the impedance, Eqs. (2) and (3) can be obtained

2 $$ Z^{\prime}\left( \omega \right) = R_{\infty } + \mathop \smallint \limits_{ - \infty }^{ + \infty } \frac{F\left( \tau \right)}{{1 + \omega^{2} \tau^{2} }}d\left( {\log \left( \tau \right)} \right), $$

and

3 $$ Z^{\prime\prime}\left( \omega \right) = \mathop \smallint \limits_{ - \infty }^{ + \infty } \frac{F\left( \tau \right)\omega \tau }{{1 + \omega^{2} \tau^{2} }}d\left( {\log \left( \tau \right)} \right), $$

where $$F\left( \tau \right) = R_{p} \cdot G\left( {\log \left( \tau \right)} \right)$$ is the DRT spectrum. Finding $$F\left( \tau \right)$$ using the above-mentioned equations is known as ill-posed inverse problem as in principle, many solutions are possible. The usage of TR is widely accepted for such problems. As solution of the right-hand sides for Eq. (2) and (3), construction of linear systems of equations is performed by discrete mesh {$$\tau_{n} ,n = 1, \ldots ,N$$}, where $$N$$ is the total number of $$f$$ points. Presently, we have used $$\tau_{n} \tau_{n} , = {\raise0.7ex\hbox{$1$} \!\mathord{\left/ {\vphantom {1 {\omega_{n} }}}\right.\kern-\nulldelimiterspace} \!\lower0.7ex\hbox{${\omega_{n} }$}}$$ and $$\omega_{n} = \tau_{n + 1} - \tau_{n}$$. Furthermore, we can modify Eqs. (2) and (3) in linear systems^15^ as

4 $$ \begin{array}{*{20}l} {\mathop \sum \limits_{n = 1}^{N} F_{n} \left( {1 + \omega_{1}^{2} \tau_{n}^{2} } \right)^{ - 1} h_{\tau } = Z^{\prime}\left( {\omega_{1} } \right) - R_{\infty } } \hfill \\ \vdots \hfill \\ {\mathop \sum \limits_{n = 1}^{N} F_{n} \left( {1 + \omega_{N}^{2} \tau_{n}^{2} } \right)^{ - 1} h_{\tau } = Z^{\prime}\left( {\omega_{n} } \right) - R_{\infty } } \hfill \\ \end{array} , $$

5 $$ \begin{array}{*{20}l} {\mathop \sum \limits_{n = 1}^{N} F_{n} \omega_{1} \tau_{n} \left( {1 + \omega_{1}^{2} \tau_{n}^{2} } \right)^{ - 1} h_{\tau } = Z^{\prime\prime}\left( {\omega_{1} } \right)} \hfill \\ \vdots \hfill \\ {\mathop \sum \limits_{n = 1}^{N} F_{n} \omega_{n} \tau_{n} \left( {1 + \omega_{n}^{2} \tau_{n}^{2} } \right)^{ - 1} h_{\tau } = Z^{\prime\prime}\left( {\omega_{n} } \right)} \hfill \\ \end{array} , $$

where $h_{\tau } = dlog\left( \tau \right)$. In terms of matrix notation $Ax = b$, $A \in {\mathbb{R}}^{m \times n} ,x \in {\mathbb{R}}^{n} ,b \in {\mathbb{R}}^{m}$. Tikhonov regularization replaces the solution by minimizing the set of equations as

6 $$ \mathop {\min }\limits_{{x\epsilon {\mathbb{R}}^{n} }} \left\{ {\left\| {Ax - b} \right\|^{2} + \lambda^{2} \left\| x \right\|^{2} } \right\}, $$

for plausible solution of the regularization parameter $\lambda$. $\left\| \cdot \right\|$ refers to the 2-norm of a matrix or vector. For any values of $\lambda > 0$ the minimization of Eq. (6) has unique solution as

7 $$ x: = \left( {A^{T} A + \lambda^{2} I} \right)^{ - 1} A^{T} b. $$

For physically acceptable solution a non-negative constraint $x_{n} \ge 0\forall n$ is applied. The $b$ matrix is constructed by both real and imaginary parts of given impedance data such as

8 $$ b = \left[ {\begin{array}{*{20}c} {Z^{\prime}\left( \omega \right)} \\ {Z^{\prime\prime}\left( \omega \right)} \\ \end{array} } \right]. $$

L-curve is a popular graphical method for determining the suitable regularization parameter. L-curve^33^ shows the negotiation between the two quantities as described in Eq. (6) and is used to find the regularization parameter. Secondly, this technique is fast and straightforward. Thirdly, computing the distribution of relaxation times is an ill-posed inverse problem and it is of utmost necessity to use some tool to reduce this deficiency. Presently, we use L-curve to find the global corner (with maximum curvature) to the residual norm. Since this does not need the information of the solution norm, provides the necessary information of the residual norm. However, offsets of the global corner are more efficient in estimating the regularization parameters as discussed in later section. The package for determining the DRTs is developed in Matlab environment and for single value decomposition Matlab based script^33^ is used. The overall code takes one to five seconds to calculate a DRT spectrum on a 1.99 GHz PC depending on the number of frequency points.

The quantification and isolation of each peak from the DRTs are done by peak fitting program assuming Gaussian distribution. A peak fitting that justifies the central position with the characteristic time constant $$\tau_{c}$$; the height, an indication of the polarization; the standard deviation, which indicates the width of the peak. Presently, the peak fitting is performed using a Matlab based script *peakfit*^34^*.* Time consumes in peak fitting also as several iterations are necessary. Overall, the polarization contribution or the $$R$$ at a characteristic time constant is equal to the normalized area under each peak multiplied by $$R_{p}$$. Then the effective capacitance ($$C_{eff}$$) is computed by $${\raise0.7ex\hbox{${\tau_{c} }$} \!\mathord{\left/ {\vphantom {{\tau_{c} } R}}\right.\kern-\nulldelimiterspace} \!\lower0.7ex\hbox{$R$}}$$ relation. Thus, we are able not only in identifying different processes but also $$R$$ and $$C_{eff}$$’s corresponding to these processes from the peaks.

## Results and discussion

In this context, impedance spectra of different electrochemical systems are analysed using the TR technique. Before processing the impedance data the quality of data has been checked by conventional KK relations using Lin-KK tool^35^.

### Single RC circuit

**Figure 1:**
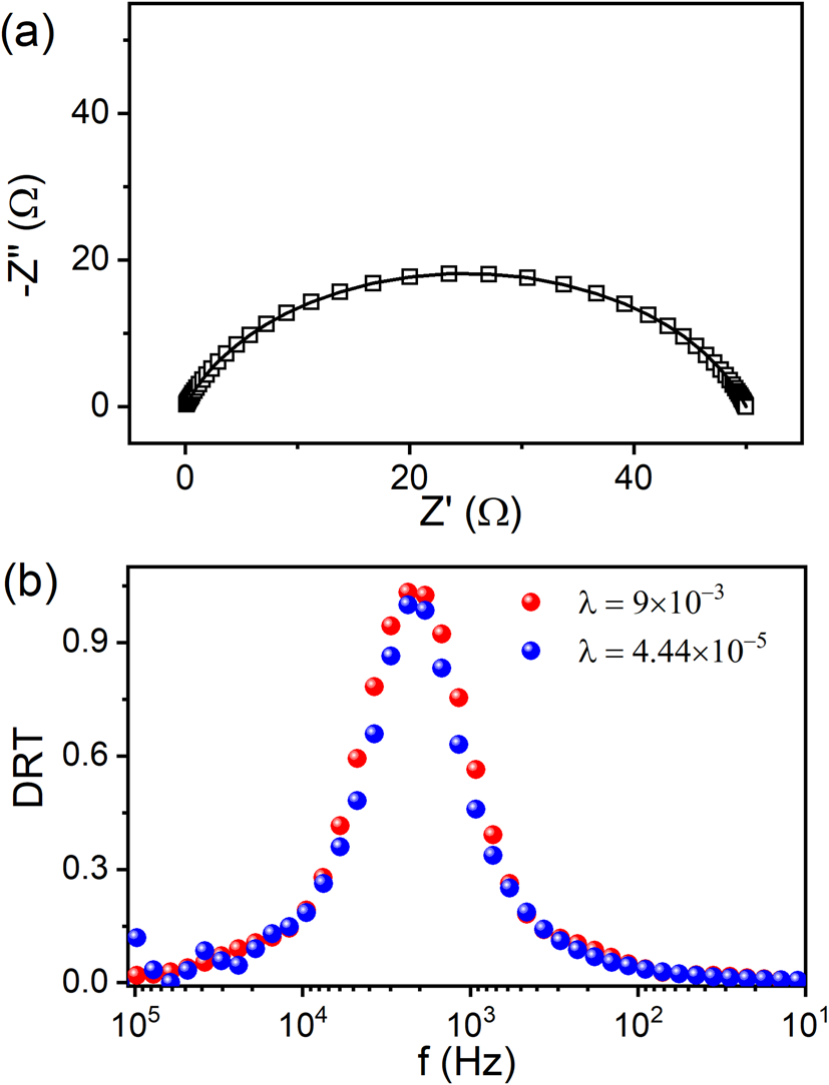
(**a**) Nyquist plots of simulated impedance and (**b**) corresponding DRT spectrum for RC circuit. The solid line is the fit using the code in (**a**).

A simulated impedance arc along with the computed arc having $$R$$ = 50 Ω and $$C$$ = 10 × 10^−6^ F are shown in Fig. 1a between 1 MHz to 1 mHz. It has been observed that the KK relations are valid in the entire frequency region (Fig. S1). The computed DRT spectrum as plotted as a function of frequency ($$f = {\raise0.7ex\hbox{$1$} \!\mathord{\left/ {\vphantom {1 {2\pi \tau }}}\right.\kern-\nulldelimiterspace} \!\lower0.7ex\hbox{${2\pi \tau }$}}$$) has a single broad peak, implying single electrochemical process (Fig. 1b). Considering both the global corner (4.44 $$\times$$ 10^−5^) and the offset of the global corner (9 $$\times$$ 10^–3^), smoother variation for the latter in the DRT especially at high frequencies is achieved (Fig. 1b). It has been observed that the global corner is not providing proper choice of a regularization parameter (Fig. S2). Similar failure of the global corner in estimation of the regularization parameter is also discussed for ill-posed inverse problem of electrical resistance tomography^36^. Nevertheless, the $$R$$ and $$C_{eff}$$ are obtained as 50.997 Ω and 1.70 × 10^–6^ F respectively from the area calculation of the peak fit considering the offset of the global corner.

### Double RC circuits

**Figure 2:**
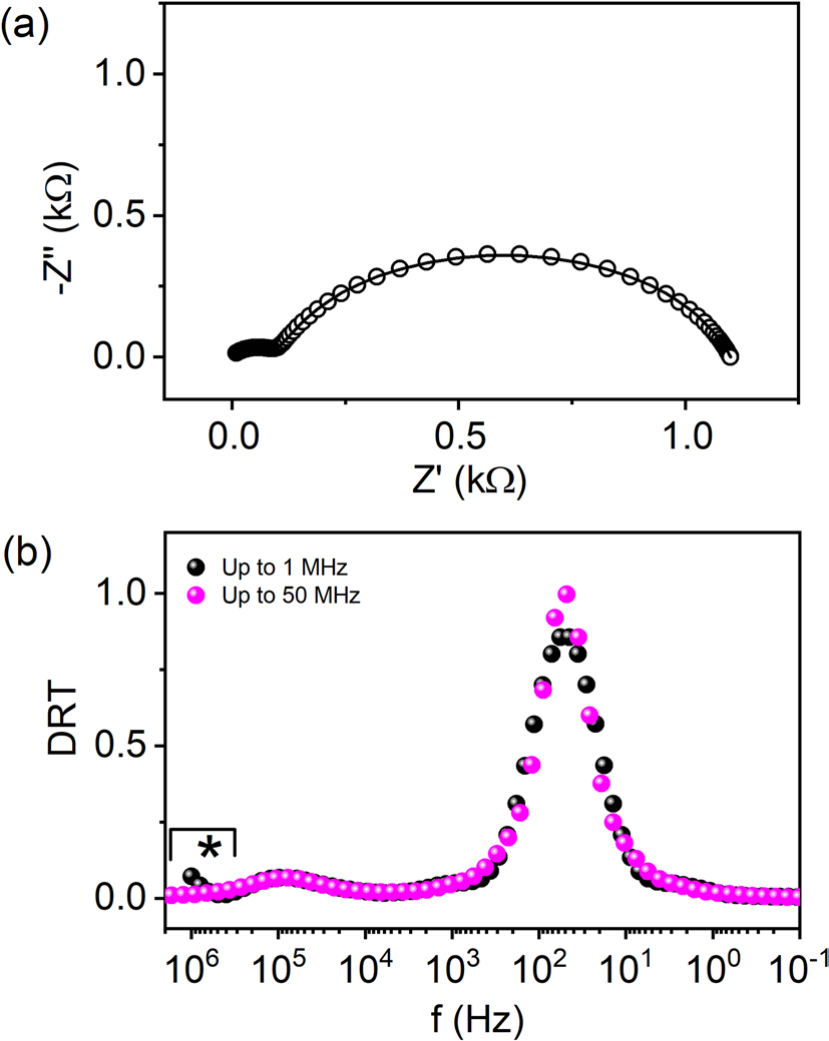
(**a**) Nyquist plots of simulated impedance and (**b**) corresponding DRT for two RC circuits. The solid line is the fit using the code in (**a**). The DRT up to 50 MHz is obtained with an offset of the global corner of 0.001 as shown in (**b**).

The simulated impedance arc for two parallel $R$ and $C$’s as connected in series having resistances and capacitances as 1 kΩ, 100 Ω and 10 × 10^–6^ F, 1 × 10^–6^ F respectively is plotted in Fig. 2a between 1 and 1 MHz. As obtained from Fig. S3, the residual errors using KK relations is null and the entire frequency range can be used for DRT calculations. Figure 2b shows the DRT spectrum corresponding to the computed impedance plot. Although global corner is obtained using the L-curve method (Fig. S4), still the DRT becomes noisy at high frequencies restricting precise estimation (Fig. S5). Further peak profile analysis leads to $R$ and $C$’s 998.82 Ω, 78.29 Ω and 3.55 × 10^–6^ F, 20.86 × 10^–9^ F respectively with $\lambda$ = 0.04 (offset of the global corner). However, we have obtained an onset of a weak pear around the marked region which is vanished when we simulate the DRT for more closed semicircle at the high frequencies (viz. 50 MHz).

### Supercapacitors

**Figure 3:**
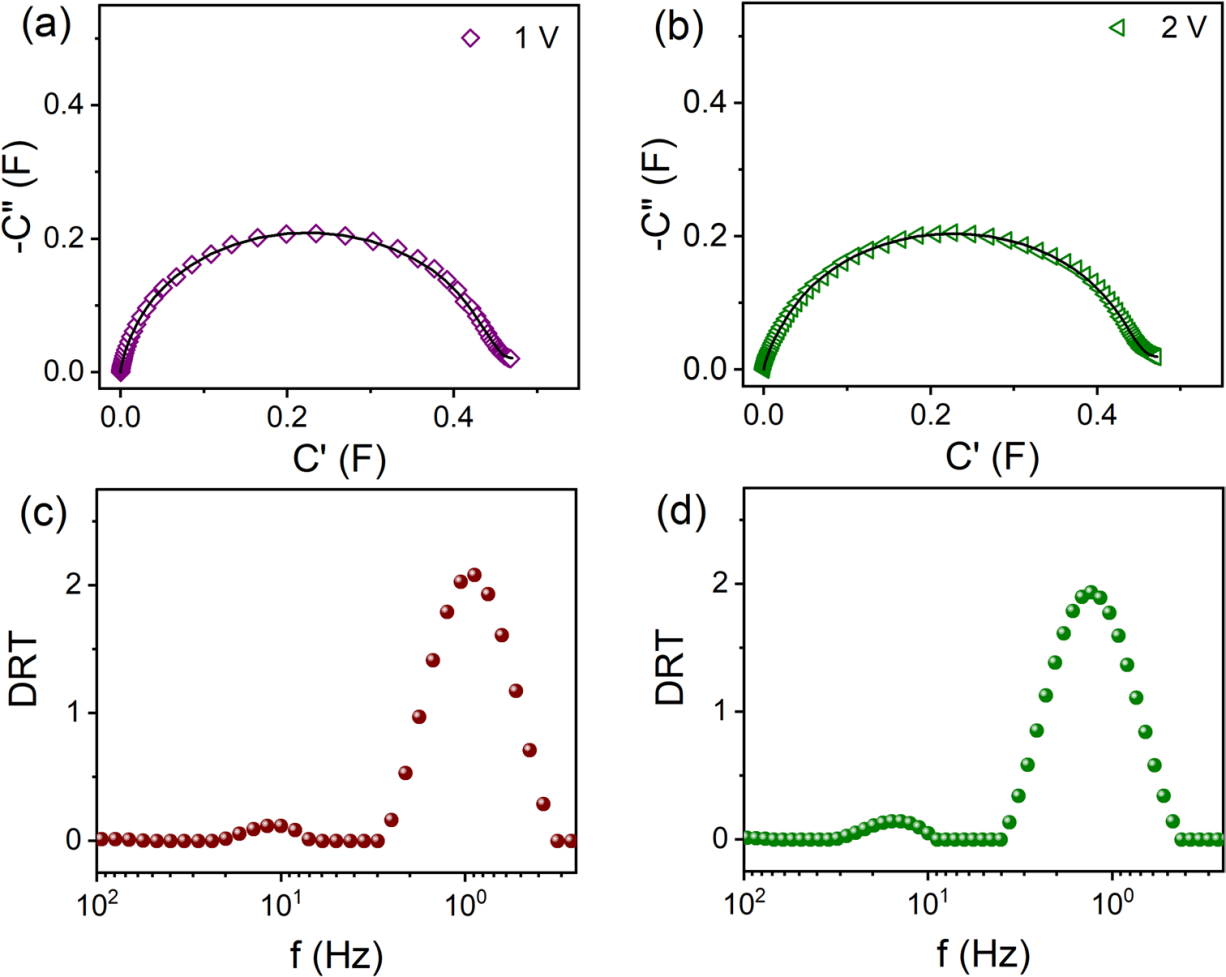
Nyquist plots of capacitances for supercapacitors at (**a**) 1 V and (**b**) 2 V dc bias voltages after 4000 cycles and corresponding DRTs are shown in (**c,d**) respectively. The solid lines in (**a,b**) are the fit using the code. The regularization parameter is set to 0.0127.

Graphite based supercapacitors have been studied electrochemically at different bias voltages with frequency range of 10 mHz to 10 kHz with an AC excitation voltage of 10 mV after 4000 cycles. To obtain a shift in the impedance plot different bias voltages are applied. It is well known that the typical Nyquist plot of impedance for supercapacitors consist of single semicircles at high to medium high frequency range but a spike like nature at low frequencies (Fig. S6). As proposed by De Levie^37^ electrical double layer capacitance (EDLC) forms towards the porous electrode at lowest frequencies and ion diffusion occurs at intermediate frequencies^38^. For the sake of completeness, the complex impedance mode has been transformed to complex capacitance mode via the relation^39,40^ $$C\left( \omega \right) = {\raise0.7ex\hbox{$1$} \!\mathord{\left/ {\vphantom {1 {j\omega Z\left( \omega \right)}}}\right.\kern-\nulldelimiterspace} \!\lower0.7ex\hbox{${j\omega Z\left( \omega \right)}$}}$$, where $$Z\left( \omega \right)$$ is the complex impedance and $$C\left( \omega \right)$$ is the complex capacitance. Here $$C^{\prime}\left( \omega \right)$$ and $$C^{\prime\prime}\left( \omega \right)$$ are the real and imaginary parts of the complex capacitance respectively (Figs. 3a,b). This transformed data show KK compatibility are within 2% as shown by the residual error plots (Figs. S7, S8). Interestingly, noise free DRTs are obtained using the regularization parameters which are obtained from the offset of the global corner of L-curve (see Figs. S9, S10). Although the impedance arcs show similar behaviour but the DRT peaks shift towards high frequency side indicating effect of charge relaxations due to increasing bias voltages (Figs. 3c,d). Presently, the total area of each peak is multiplied by the normalization factor to obtain $$C_{eff}$$. The DRTs are comprised of two broad peaks around 10 Hz and 1 Hz with capacitances 0.06 F, 0.57 F, and 0.08 F, 0.57 F, for 1 V and 2 V respectively. The peaks around 10 Hz and 1 Hz can be associated with diffusion of electrolyte ions towards and inside the pores of the electrodes and EDLC respectively^40^. The diffusion process consumes maximum capacitance due to local polarizations^40^.

### Li-ion battery

**Figure 4:**
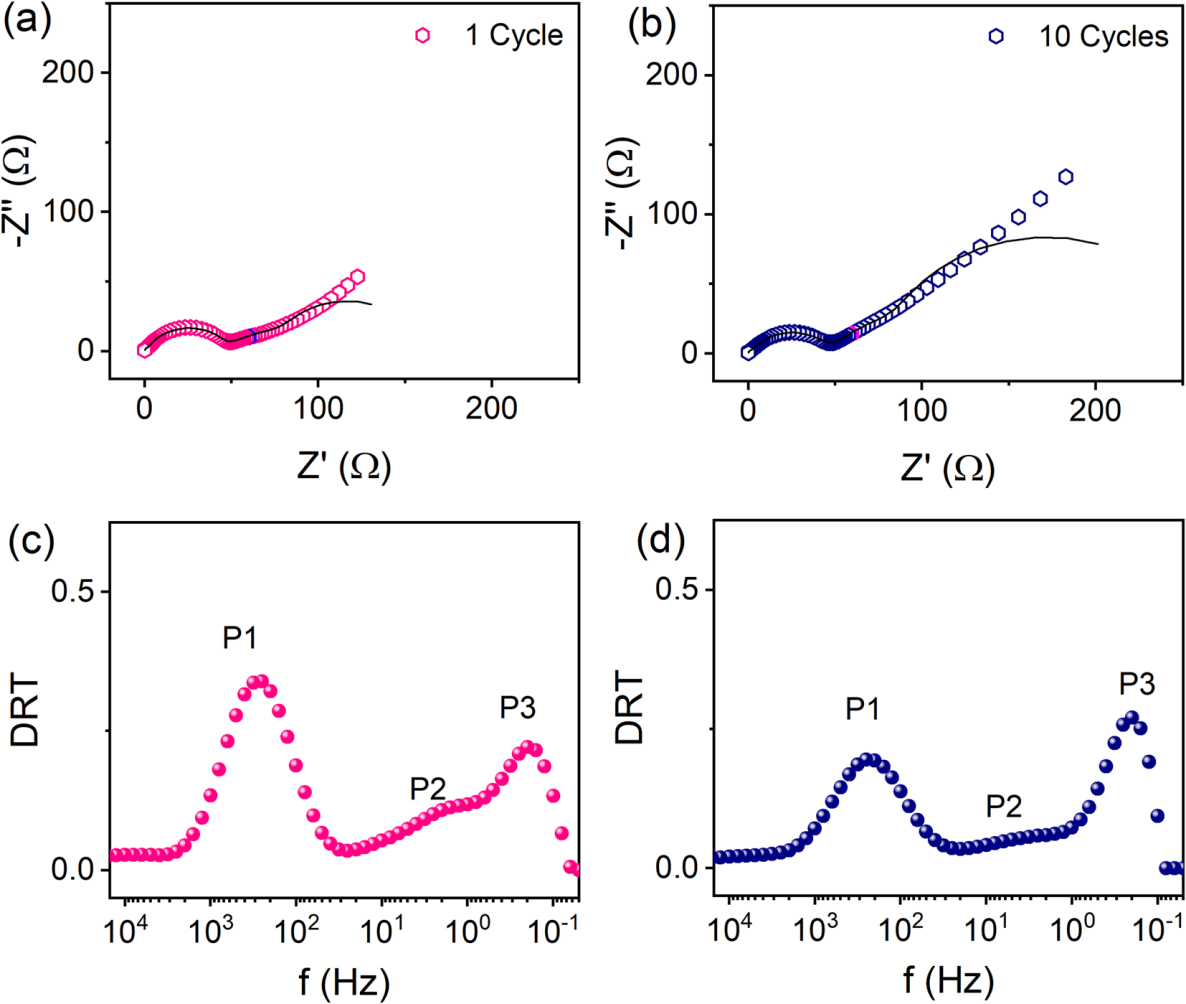
Nyquist plots of impedances from LIBs after (**a**) 1 cycle and (**b**) 10 cycles of charge/discharging and corresponding DRTs are shown in (**c,d**) respectively. The solid lines in (**a,b**) and (**b**) are the fit using the code. The solid symbol at 1 Hz is also marked in both (**a,b**).

Lithium ion batteries (LIB) show complex nature in their impedance spectra due to different electrochemical processes. In the half cell configuration of LIBs, impedance response consists of contributions from anode with its solid electrolyte interphase, the cathode and electrolyte having polarization processes like ohmic losses, charge transfer processes, as well as diffusion processes^41^. Presently the system under study is LIB coin cells with the cell configuration of α-LiFeO_2_/LiPF_6_/Li where the electrolyte is mixture of LiPF_6_ in ethylene carbonate (EC) and dimethyl carbonate (DMC) having 1:1 volume ratio. The impedance data have been measured at room temperature with a minimum frequency of 10 mHz up to maximum frequency of 100 kHz with 71 measured frequencies in total. The AC excitation voltage was set as 10 mV, and the impedance data have been obtained after 1st, 5th and 10th cycles of cyclic voltammetry measurements. The total impedance increases with increasing cycling as observed in Figs. 4a,b. Since we have observed a spike like extension towards $\omega \to 0$ for $Z^{\prime\prime}\left( \omega \right)$ vs $f$, (not shown) restricting the use of DRTs at low frequencies for LIBs. On the other hand, at present, the KK relations are valid up to 1 Hz for the impedance data, restricting the computation of the DRTs up to 1 Hz from high frequencies (Figs. S11, S12, residue errors are within 1%). This also explains why the fitting deviates significantly at low frequencies. As observed from Figs. S13 and S14 the global corner does not produce satisfactory DRT. Henceforth, we have used the offset of the global corner as a regularization parameter (λ = 0.0659) (Figs. S13, S15–S17). The calculated DRTs consist of three peaks as shown in Fig. 4c, Supplemental Fig. S18, and Fig. 4d after 1st, 5th and 10th cycles of cyclic voltammetry measurements respectively. The ohmic resistances are subtracted before the DRT calculations, thus the impedance spectra consist of polarization behaviour only. Furthermore, peak fitting analysis suggests that three peaks are located around 251 Hz, 2 Hz and 0.20 Hz named as P1, P2 and P3 respectively (Fig. 4c,d, Supplemental Fig. S19). With increasing cycles, the effective resistances for peaks P1 and P2 decrease while for P3 increases (see Table 1). Concerning the positions of the peaks and resistance values peaks P1, P2 and P3 correspond to surface film resistance as originated due to Li ion migration through the electrode surfaces, and α-LiFeO_2_/electrolyte interfacial charge transfer resistances respectively^42^. It is noted, DRT analysis has been investigated on LiFePO_4_/lithium cells^9^. The results show solid state diffusion in the cathode, and charge transfer resistance between cathode and electrolyte in between 10^3^ and 1 Hz at different temperatures and state of charge^9^. The spike like extension at low frequency regime is avoided by pre-processing using equivalent circuit model in Ref.^9^. In our study, we have neglected this regime indirectly by choosing the KK compatible regime only. In this way, we have considered only the polarization behaviour and identified anode related phenomena as shown in Fig. 4c,d. As our interest lies within the KK compatible regime and for Li-ion batteries the electrochemical phenomena occur within the semicircular regime only. Considering these facts peak P1 and P2 are of utmost interest and since peak P3 is in the vicinity of the incompatible regime lower confidence level is obtained.

**Table 1:** Table for effective resistances and capacitances calculated from the DRTs of Fig. 4.

Condition	Peak P1		Peak P2		Peak P3	
	R (Ω)	C (F)	R (Ω)	C (F)	R (Ω)	C (F)
1 cycle	38.47	1.49 × 10^−5^	17.76	0.0074	12.21	0.068
5 cycles	38.42	1.65 × 10^−5^	20.07	0.0049	24.22	0.029
10 cycles	35.05	1.95 × 10^−5^	14.98	0.0072	25.48	0.028

**Figure 5:**
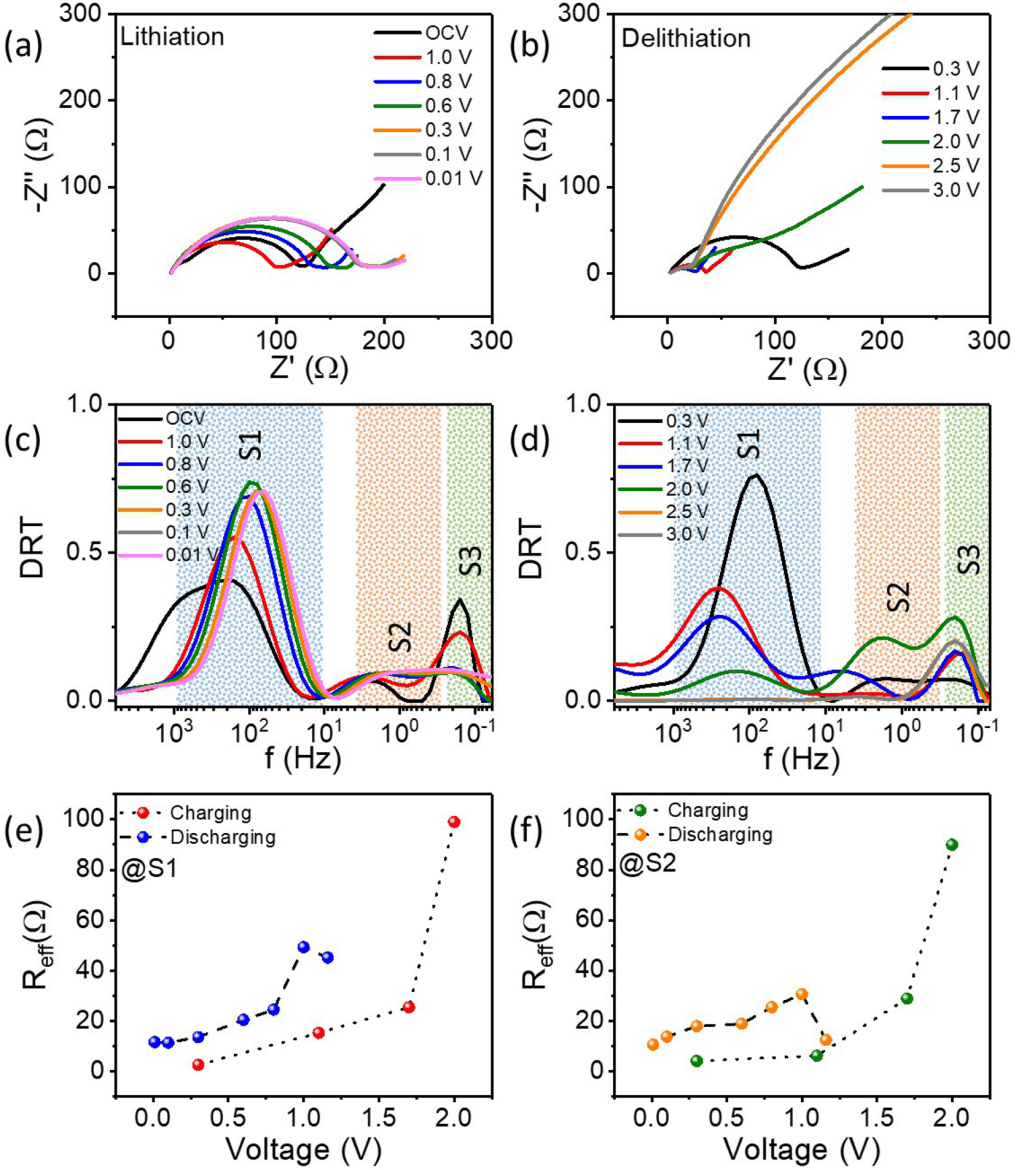
Nyquist plots of impedances from LIBs during (**a**) 1st discharging and (**b**) 1st charging and corresponding DRTs are shown in (**c,d**) respectively with the offset of the global corner as a regularization parameter (λ = 0.06334). The shadings in (**c,d**) are for illustration only. The voltage dependence of the effective resistances as obtained for peak S1 and S2 are shown in (**e,f**) respectively. The dash lines in (**e,f**) are guide to the eye.

As observed from Fig. S20, during the first discharge curve a sharp peak at 0.56 V attributes to the formation of solid electrolyte interface^43^ as well as reduction of Fe^2+^ and Fe^3+^ to Fe^0^ Ref.^44^. Secondly, during charging, a broad peak around 1.69 V corresponds to oxidation of Fe^0^ to Fe^3+^ Ref.^45^. To understand the effect of the lithiation and delithiation peaks on the ion transport additional EIS measurements were performed at 0.1 °C rate at different potentials and the results are shown in Fig. 5a,b. Distinguished features in the semicircular arcs as well as in the tails at low frequencies are noted for both charging and discharging potentials. Additionally, the DRTs are shown in Fig. 5c,d considering the offset of the global corner of the L-curve method (see Fig. S21). To better understand our observation, we have also determined the DRT of Li||Li symmetric cell as shown in Fig. S22. Comparing Fig. S22 with Fig. 5c,d, we can conclude that the peak S1 (between 10^3^ and 10 Hz) is related to Li metal to electrolyte interface and continues till 2.5 V of charging as observed in Fig. 5d. Interestingly, during discharging, peak S1 decreases towards low frequency, whereas it shifts towards high frequency during charging exhibiting the effect of lithiation and delithiation respectively, which are also time bound processes. It can be concluded that these potentials activate the lithium related process but the direction of the potential sweep (OCV (1.16 V) → 0.01 V → 3 V) corresponds to the shifting of the peak position. An increasing trend in their effective resistances exhibits the aging effect as displayed in Fig. 5e. In addition, the peaks S2 and S3 occurring between 10 and 0.1 Hz correspond to Li ion motion for the α-LiFeO_2_/electrolyte interfacial charge transfer process, and the solid state Li ion diffusion respectively. Similar to the previous description, peak S3 is in the KK incompatible regime so that we do not carry out further analysis. The lithiation at 0.46 V (Fig. S20) has strong effect on peak S2 both in position as well as in peak shape. For example, it is observed that initially at OCV and 1 V of discharging the S2 is separable from S3 and not visible beyond 2 V during charging of the battery (Fig. 5 (c) and (d)). Since S2 evolves from the electrode/electrolyte interface as well as direction dependent of the potential sweep (OCV → 0.01 V → 3 V), it is expected to be related to the reduction and oxidation of Fe respectively. The effective resistance related to peak S2 also shows increasing behaviour as shown in Fig. 5f. It is noted that the reduction of Fe^3+^ and Fe^2+^ to Fe^0^ is related to the initial capacity drop in ferrite based LIBs^44^. Therefore, our results provide clues to better understand not only the different electrochemical processes, but also the different transitions of Fe in the α-LiFeO_2_ electrode.

## Conclusions

In this paper, a Matlab based fast application is developed to transform the EIS data into non-parametric DRT arrangement. The code has been developed with the help of Tikhonov regularization with special constraint for eliminating non-negative parts in DRTs. The global corner of L-curve is failed to compute meaningful DRTs whereas the offset of the global corner is found to be useful. Nevertheless, we are successful in identifying different electrochemical processes from the DRTs by peak fitting methods quantitatively. For instance, electrical double layer capacitance and ion diffusion phenomena are identified for supercapacitors in low to intermediate frequencies respectively. Moreover, we have used the method to identify the three processes contributing to the half-cell LIBs. These processes are related to the charge transfer reactions and surface reaction processes. More detailed analysis of the EIS data taken at different potentials guided from CV data provide clues to better understand the correlation between the capacity fading in battery cycling and Li/electrolyte and electrode/electrolyte charge transfer related processes.

## Supplementary Information

Supplementary Information.

## Data Availability

The data that support the findings of this study are available from the corresponding authors upon request. The code is available from https://github.com/paultanmoy00/Impedance-modelling/.
