# Supplementary material for: Computation of distribution of relaxation times by Tikhonov regularization for Li ion batteries: usage of L-curve method
Source: Sci Rep. 2021 Jun 16;11:12624. doi: 10.1038/s41598-021-91871-3 (PMC8209233; doi:10.1038/s41598-021-91871-3)
Supplement: Supplementary file 1 — Supplementary Information. [file 41598_2021_91871_MOESM1_ESM.docx]

**Computation of Distribution of Relaxation Times by Tikhonov Regularization for Li ion batteries: Usage of L-curve method**

T. Paul^1*^, P. W. Chi^1^, Phillip M. Wu^1,2,3*^, M. K. Wu^1^

^1^Institute of Physics, Academia Sinica, Taipei, Taiwan

^2^BitSmart LLC, San Mateo, CA, USA

^3^Present address: Department of Materials and Mineral Resources Engineering, National Taipei University of Technology, 1, Sec. 3, Zhong_xiao E. Rd, Taipei, 10608, Taiwan

*Email: [paultanmoy00@gmail.com](mailto:paultanmoy00@gmail.com) (T. Paul); [philwu@gmail.com](mailto:philwu@gmail.com) (P. M. Wu)


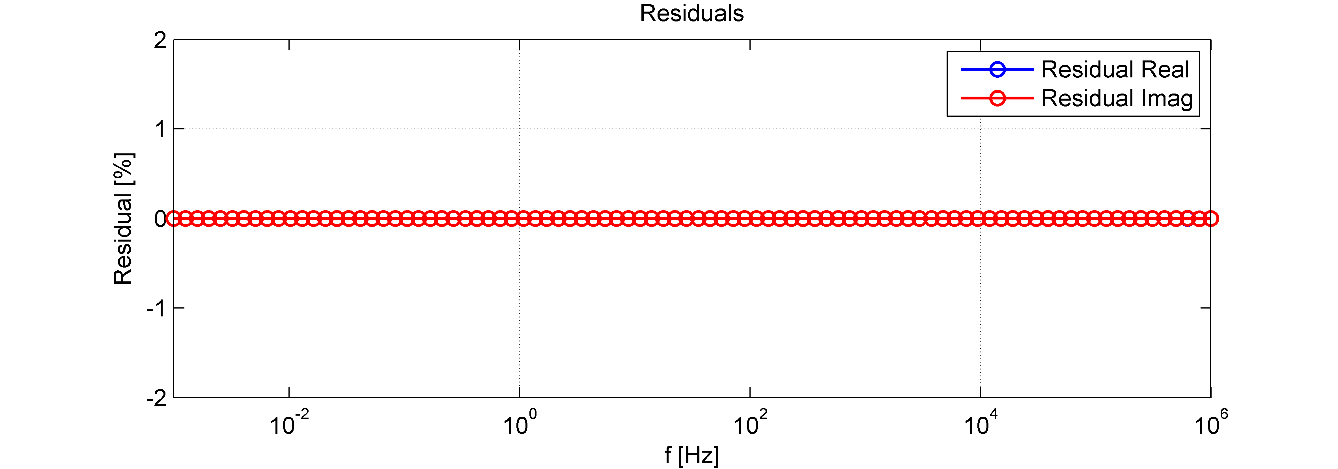


Fig. S1. Residual error as calculated using KK-relations for single RC circuit.

Fig. S2. L-curve for single RC circuit to determine λ from the corner of the graph. The dotted line shows the global corner. The offset region is also marked.


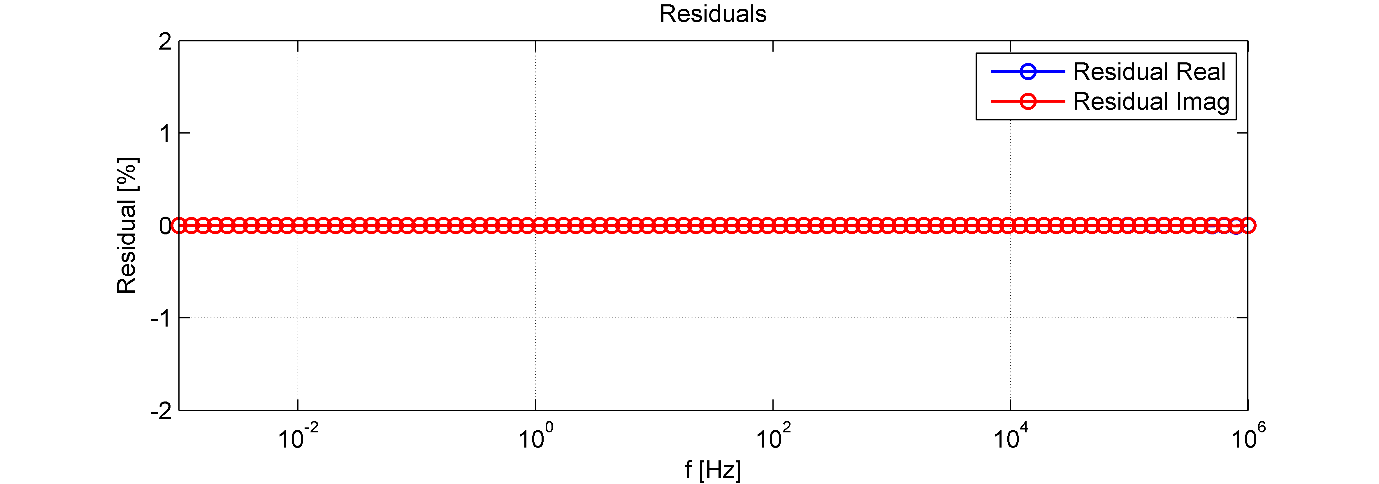


Fig. S3. Residual error as calculated using KK-relations for double RC circuits with series connection.

Fig. S4. L-curve for 2 RC circuits with different capacitances and resistances in series to determine λ. The dotted line shows the global corner.

Fig. S5. The DRT as calculated from the above-mentioned λ indicating oscillatory behaviour at high frequencies.





Fig. S6. Nyquist plot of impedance response for supercapcitor obtained at 2 V after 4000 cycles.


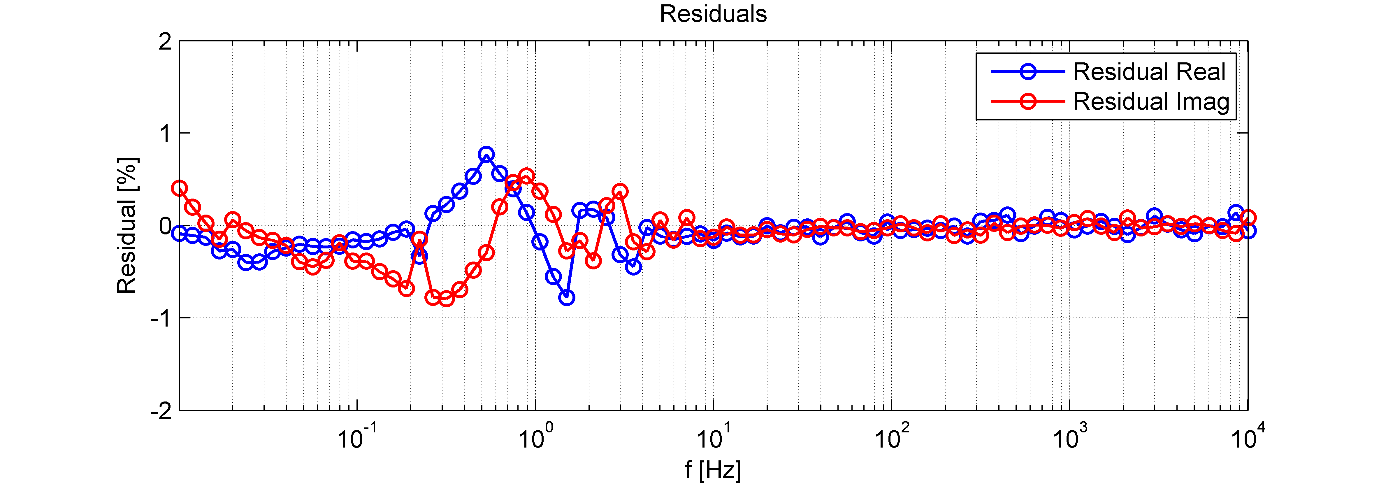


Fig. S7. Residual errors as calculated using KK-relations for supercapacitor after running 4000 cycles at 1V.


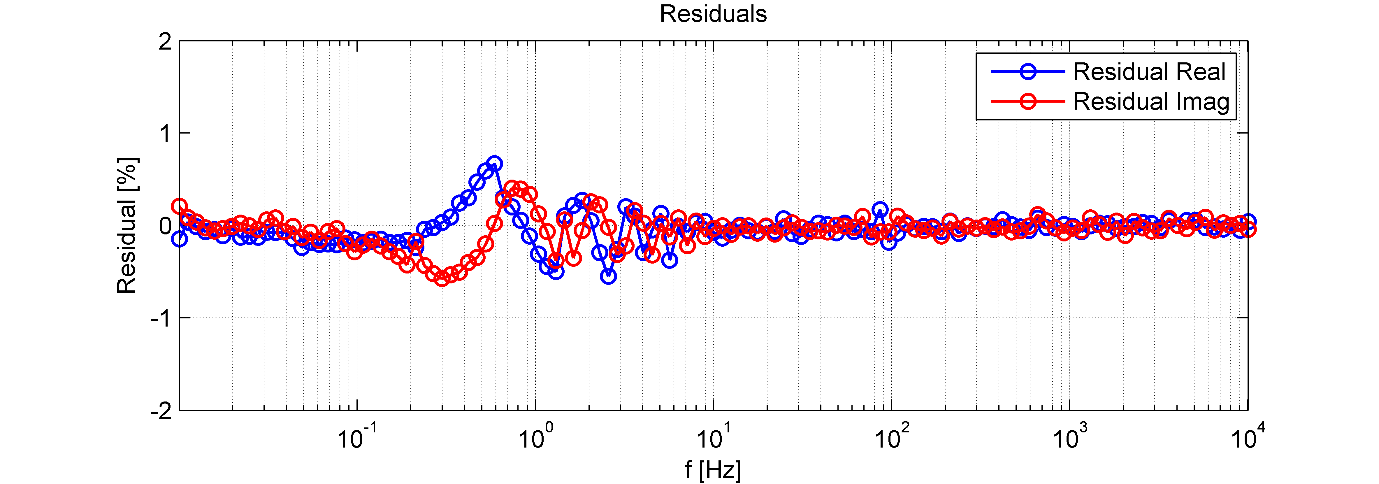


Fig. S8. Residual errors as calculated using KK-relations for supercapacitor after running 4000 cycles at 2V.

Fig. S9. L-curve with its corner for determining λ after running 4000 cycles at 1V for supercapacitor. The dotted line shows the global corner.

Fig. S10. L-curve with its global corner for determining λ after running 4000 cycles at 2V for supercapacitor. The dotted line shows the global corner.


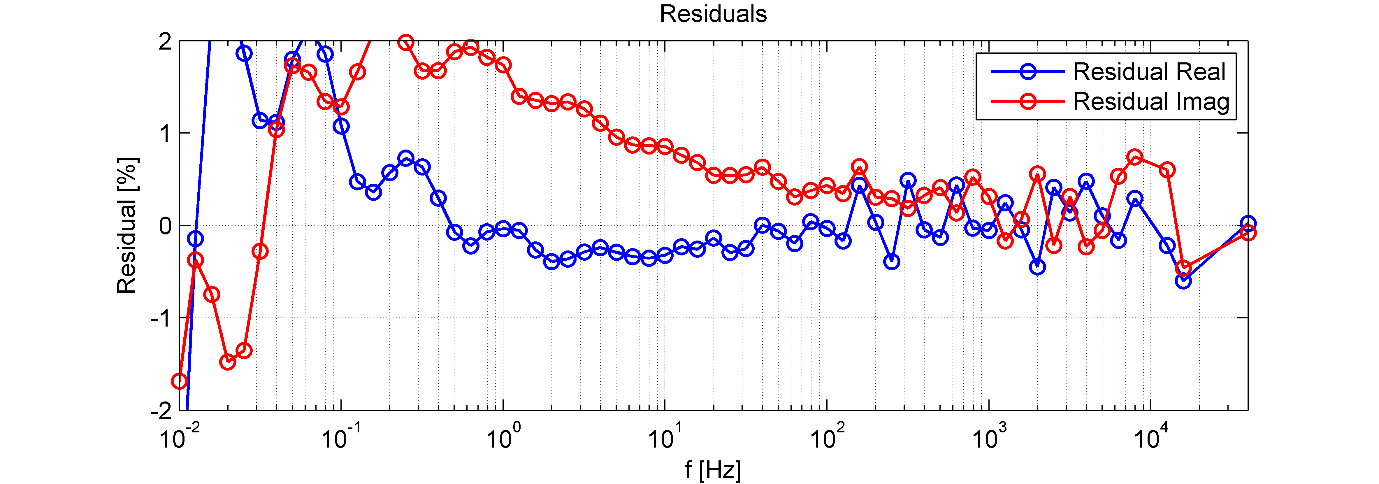


Fig. S11. Residual errors as calculated using KK-relations after running 1 CV measurement for LIB.


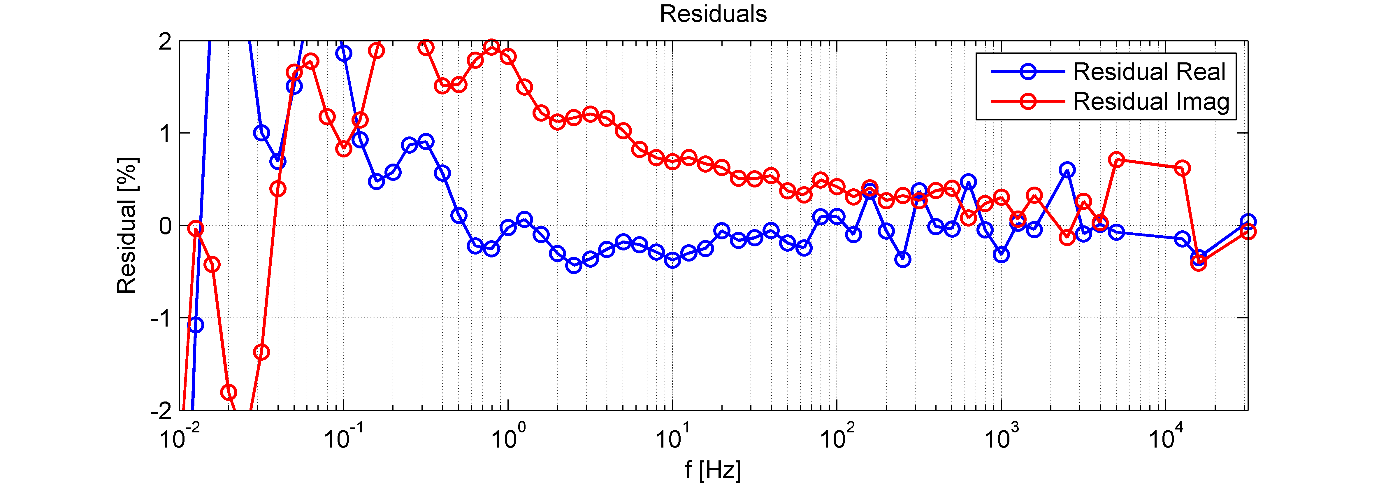


Fig. S12. Residual errors as calculated using KK-relations after running 10 CV measurements for LIB.

Fig. S13. L-curve to determine L-corner for half-cell configuration of Li ion battery after 1 CV measurement. The global corner is obtained at 0.00079049.

Fig. S14. DRT as obtained with the global corner as a regularization parameter after 1 CV cycle measurement.

Fig. S15. L-curve to determine L-corner for half-cell configuration of Li ion battery after 1 CV measurement with offset of global corner. The dotted line shows the offset of the global corner.

Fig. S16. L-curve to determine global corner for half-cell configuration of Li ion battery after 5 CV measurements. The dotted line shows the global corner.

Fig. S17. L-curve to determine global L-corner for half-cell configuration of Li ion battery after 10 CV measurements. The dotted line shows the global corner.





Fig. S18. The DRT after 5^th^ cycle for LIB is shown with the offset of the regularization parameter.





Fig. S19. Deconvolution of DRT peaks for LIB after 1 CV cycle measurement.





Fig. S20. Cyclic Voltammetry curve during 1^st^ cycle for α-LiFeO_2_ anode based LIB.

Fig. S21. L-curve to determine global L-corner for half-cell configuration of Li ion battery operated at 2.5 V of charging. The dotted line shows the global corner.


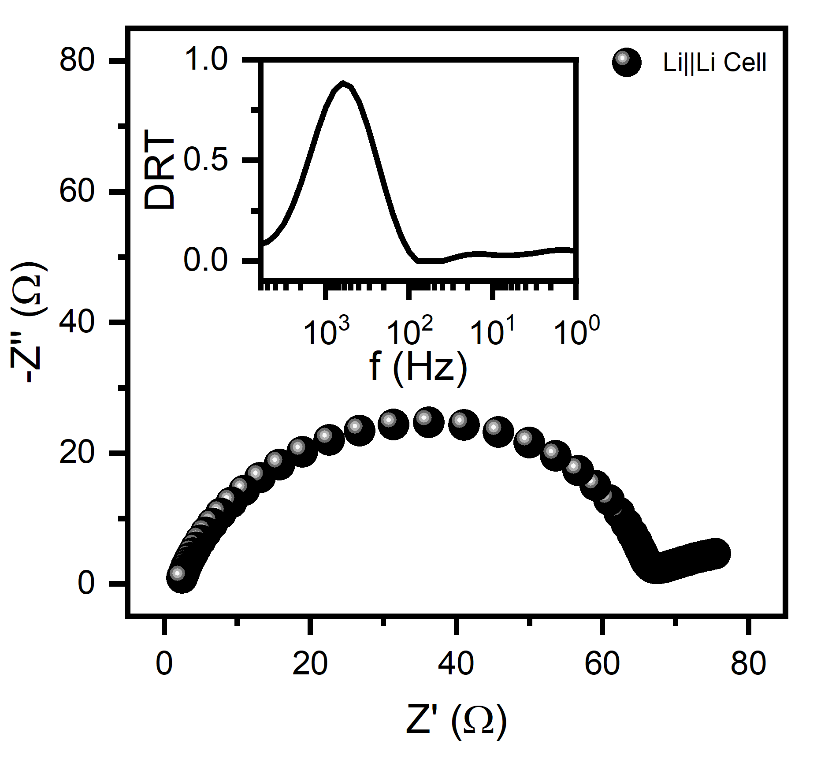


Fig. S22. Nyquist plot of impedance and in the inset the corresponding DRT for Li||Li symmetric cell. The DRT is obtained with the offset of the global corner as a regularization parameter (λ = 0.056775).
